# Serum calprotectin (S100A8/9): an independent predictor of ultrasound synovitis in patients with rheumatoid arthritis

**DOI:** 10.1186/s13075-015-0764-5

**Published:** 2015-09-15

**Authors:** Jana Hurnakova, Jakub Zavada, Petra Hanova, Hana Hulejova, Martin Klein, Herman Mann, Olga Sleglova, Marta Olejarova, Sarka Forejtova, Olga Ruzickova, Martin Komarc, Jiri Vencovsky, Karel Pavelka, Ladislav Senolt

**Affiliations:** Institute of Rheumatology, Na Slupi 4, 128 05 Prague 2, Czech Republic; Department of Rheumatology, 1st Faculty of Medicine, Charles University, Prague, Czech Republic; Institute of biophysics and informatics, 1st Faculty of Medicine, Charles University, Prague, Czech Republic

## Abstract

**Introduction:**

Calprotectin, a heterodimeric complex of S100A8/9 (MRP8/14), has been proposed as an important serum biomarker that reflects disease activity and structural joint damage in rheumatoid arthritis (RA). The objective of this cross-sectional study was to test the hypothesis that calprotectin is associated with clinical and ultrasound-determined disease activity in patients with RA.

**Methods:**

A total of 37 patients with RA (including 24 females, a mean disease duration of 20 months) underwent a clinical examination and 7-joint ultrasound score (German US-7) of the clinically dominant hand and foot to assess synovitis by grey-scale (GS) and synovial vascularity by power Doppler (PD) ultrasound using semiquantitative 0–3 grading. The levels of serum calprotectin and C-reactive protein (CRP) and erythrocyte sedimentation rate (ESR) were determined at the time of the ultrasound assessment. We analysed the relationship between serum calprotectin level, traditional inflammatory markers, and ultrasound-determined synovitis.

**Results:**

The levels of serum calprotectin were significantly correlated with swollen joint count (r = 0.465, p < 0.005), DAS28-ESR (r = 0.430, p < 0.01), ESR (r = 0.370, p < 0.05) and, in particular, CRP (r = 0.629, p < 0.001). Calprotectin was significantly associated with GS (r = 0.359, p < 0.05) and PD synovitis scores (r = 0.497, p < 0.005). Using multivariate regression analysis, calprotectin, adjusted for age and sex, was a better predictor of PD synovitis score (R^2^ = 0.765, p < 0.001) than CRP (R^2^ = 0.496, p < 0.001).

**Conclusions:**

The serum levels of calprotectin are significantly associated with clinical, laboratory and ultrasound assessments of RA disease activity. These results suggest that calprotectin might be superior to CRP for monitoring ultrasound-determined synovial inflammation in RA patients.

## Introduction

Rheumatoid arthritis (RA) is a common chronic inflammatory autoimmune disease characterised by persistent synovitis, the development of joint deformities, the presence of autoantibodies and an increased risk of cardiovascular comorbidities [1]. Recent data clearly suggest that treatment to target is an optimal treatment strategy providing the best results for suppressing inflammation and thus avoiding irreversible joint damage in patients with RA [2, 3]. In contrast to the majority of chronic diseases such as hyperlipidaemia or diabetes mellitus, there is no single gold standard biomarker for RA. A physical examination of patients’ joints and the parameters of the acute-phase response are routinely used in daily clinical practice to assess the disease activity of RA. The target of treatment in RA is remission or low disease activity, which is obtained by using several composite indexes [4–7]. Nevertheless, a clinical examination might lack sensitivity in patients with mild synovitis, be limited in patients with established deformities, and be overestimated in patients with concomitant fibromyalgia or other comorbidities. Therefore, identifying novel, sensitive serum biomarkers of RA disease activity remains challenging.

Calprotectin is a heterodimeric complex of S100A8/A9 [8] (myeloid-related protein, MRP8/MRP14) [9], calgranulin A and B [10], L1 protein [11] and cystic fibrosis antigen [12]. It is released during inflammatory processes, predominantly from activated leukocytes at the sites of joint inflammation [13], and could be used as a valuable biomarker of several inflammatory diseases, including RA [14–19]. Calprotectin is upregulated in RA synovial fluid and synovial tissue, with a large amount of the protein in the immune cells adjacent to the sites of joint damage [20]. Circulating calprotectin is elevated in active disease and decreases after effective treatment; it has been shown to potentially be a more sensitive biomarker of disease activity in RA than conventionally used acute-phase proteins predominantly of hepatic origin [21]. High calprotectin levels have been demonstrated to predict radiographically determined progression of RA [22].

Musculoskeletal ultrasonography is used increasingly as a valuable imaging tool for evaluating RA activity [23]. Numerous studies have shown that the sensitivity of ultrasound examination is superior to that of clinical examination [24–26], including the determination of subclinical synovitis [27–32]. Hammer et al. [33] recently demonstrated in a small study that circulating calprotectin is significantly associated with ultrasound disease activity in patients with RA. Therefore, the aim of this study was to compare the association between calprotectin, traditional markers of inflammation and ultrasound-determined RA disease activity.

## Methods

### Patients

Thirty-seven patients fulfilling the American College of Rheumatologists (ACR) 1987 [34] and/or the ACR/European League against Rheumatism (EULAR) 2010 classification criteria for RA [35] were recruited for this study. All the patients were recruited from the outpatient rheumatology clinic at the Institute of Rheumatology in Prague. The study was performed according to the principles of the Declaration of Helsinki and was approved by the local ethics committee of the Institute of Rheumatology in Prague. Informed consent was obtained from all of the patients before entry into the study. The patient characteristics are provided in Table 1. All of the patients underwent an assessment of tender and swollen joints by an experienced study nurse. Disease activity was assessed by the disease activity score for 28 joints (DAS28), using the swollen joint count (SJC) and tender joint count (TJC), erythrocyte sedimentation rate (ESR), and the patient’s global assessment of activity on a visual analogue scale (VAS).

**Table 1:** Baseline characteristics of the patients with rheumatoid arthritis

Characteristics	Rheumatoid arthritis patients (n = 37)
Female (%)	24 (64.9 %)
Age, years	53.56 ± 16.89
RF positivity, n (%)	17 (56.8 %)
Anti-CCP positivity, n (%)	22 (59.5 %)
Calprotectin, mg/L	3.91 ± 5.86
CRP, mg/L	7.55 ± 11.54
ESR, mm/1^st^ hour	16.5 ± 12.37
DAS28-ESR	3.27 ± 1.6
GS syn score (0–39)	3.78 ± 4.24
PD syn score (0–39)	2.19 ± 3.49
GS ten score (0–5)	0.27 ± 0.61
PD ten score (0–15)	0.24 ± 0.68

The values are the mean ± SD (range), if not stated otherwise. *RF* rheumatoid factor, *Anti*-*CCP* anticyclic citrullinated peptide antibody, *CRP* C-reactive protein, *ESR* erythrocyte sedimentation rate, *DAS28*-*ESR* Disease activity score for 28 joints with ESR, *GS syn* Grey scale synovitis, *PD syn* Power Doppler synovitis, *GS ten* Grey scale tenosynovitis, *PD ten* Power Doppler tenosynovitis

### Laboratory assessment

Fasting blood samples were obtained on the same day that the ultrasound examination was performed. The serum samples were centrifuged and stored at −80 °C until the analysis. Calprotectin was measured by a commercially available enzyme-linked immunosorbent assay (ELISA) according to the manufacturer’s instructional protocol (Bühlmann Laboratories AG, Schőnenbuch, Switzerland). The inter-assay and intra-assay reliability of the S100A8/9 assays were 5.8 % and 4.3 %, respectively, and the detection limits were 0.4 μg/mL. ESR was measured using a BD-15^TM^ instrument (BD, NJ, USA). C-reactive protein (CRP) level was measured using turbidimetry (Beckman (Beckman Coulter, CA, USA). Anti-cyclic citrullinated peptide (anti-CCP) antibodies were analysed using standard ELISA kits (Test Line s.r.o., Brno, Czech Republic).

### Ultrasound imaging

The ultrasound examinations were performed with Esaote Mylab 60 equipment (Esaote S.p.A., Genova, Italy) using a linear transducer with a 18 MHz frequency. The Power Doppler was pre-set, and no adjustments of the Doppler parameters were allowed. The patients were examined according to the German US7 score in the following seven joint areas: wrist, second and third metacarpophalangeal (MCP) and second and third proximal interphalangeal (PIP) and second and fifth metatarsophalangeal joints of the clinically more affected hand and foot. [36]. We used a modification of the original German US7 [37]. In contrast to the original US7, which examines synovitis of the MCP and PIP joints in Grey scale (GS) only from the palmar view, we assessed synovitis in GS in this area using both the palmar and the dorsal view. Further, in contrast to the original German US7, which assesses tenosynovitis/paratenonitis on the second and third finger both from palmar and dorsal aspect, we assessed tenosynovitis only from the palmar aspect. Synovitis in the GS was scored semiquantitatively (0 = absence, 1 = mild, 2 = moderate, 3 = severe synovitis), as follows: grade 1 = a small hypoechoic/anechoic line beneath the joint capsule; grade 2 = the joint capsule elevated parallel to the joint area; and grade 3 = a strong distension of the joint capsule [38]. Synovitis and tenosynovitis were classified semiquantitatively by power Doppler (PD), as follows: grade 0 = no intraarticular colour signal; grade 1 = up to three single colour signals or two single signals and one confluent signal in the intraarticular area; grade 2 = greater than grade 1 to <50 % of the intraarticular area filled with colour signals; and grade 3 = ≥50 % of the intra-articular area filled with colour signals. Tenosynovitis in the GS was registered as absent (0) or present (1). An overall GS and PD signal score was calculated as the sum of GS synovitis, PD synovitis and GS tenosynovitis and PD tenosynovitis with the scoring range 0–39 for GS synovitis, 0–39 for PD synovitis, 0–5 for GS tenosynovitis and 0–15 for PD tenosynovitis. The ultrasonographers were unaware of each patient’s clinical examination and laboratory findings.

To test the ultrasound interobserver reliability, eight intermediate-level or advanced-level ultrasonography investigators performed an ultrasound examination, using the US7 German score, in five RA patients with various degrees of disease activity. After 24 hours, a re-examination was performed to assess the intraobserver reliability.

### Statistical analysis

The data are described as the mean and SD, unless stated otherwise. Basic descriptive statistics (the mean, median, SD, skewness and kurtosis) were computed for all of the variables, which were subsequently tested for normal distribution using the Kolmogorov-Smirnov test. The bivariate relationship between the variables was assessed using Spearman’s correlation coefficient. Univariate and multivariate regression analyses were used to predict PD synovitis by a set of predictors (calprotectin and CRP), adjusted for age and sex. *P* values less than 0.05 were considered statistically significant. The interobserver agreement between readers and intraobserver agreement between repeat readings by the same observer were calculated using non-weighted Cohen’s kappa coefficients. The kappa coefficients were assessed according to the convention suggested by Landis and Koch, who characterise values <0 as an indication of having no agreement, 0–0.20 as having poor agreement, 0.21–0.40 as having fair agreement, 0.41–0.60 as having moderate agreement, 0.61–0.80 as having substantial agreement, and 0.81–1.0 as having excellent agreement [39]. The agreement percentages were calculated. The statistical analysis was performed using SPSS version 17 statistical software (SPSS, Inc., Chicago, IL, USA).

## Results

### Baseline patient characteristics

The clinical, laboratory and ultrasound characteristics of the patients are shown in Table 1. At the time of examination, four patients had highly active disease (DAS28 >5.1), 10 patients had moderate disease activity (3.2< DAS28 ≤5.1), four patients had low disease activity (2.6≤ DAS28 <3.2) and 19 patients were in remission (DAS28 <2.6). Rheumatoid factor (RF) and anti-CCP positivity were found in 57 % and 60 % of RA patients, respectively.

The mean (SD) symptom duration was 20 (15) months from the initial clinical symptoms. Twenty-seven patients were treated with methotrexate (at a mean dose of 13.6 mg/week, range 5 to 20 mg/week), two patients were treated with sulfasalazine (at a mean daily dose of 2.5 g, range 2 to 3 g), two patients were disease-modifying anti-rheumatic drug (DMARD)-naive, and four patients in stable remission were without treatment. Nine patients received glucocorticoids (at a mean daily dose of 11.25 mg of prednisolone or its equivalent, range 2.5 to 20 mg).

### Ultrasound interobserver and intraobserver reliability

The overall interobserver agreement among the eight investigators performing US7 in the five RA patients in two rounds was 0.607, which indicates borderline moderate to substantial agreement. Substantial interobserver agreement (kappa = 0.717) was found for synovitis in GS, and moderate inter-reader agreement was found for synovitis in PD (kappa = 0.527). The mean intraobserver agreement was excellent (kappa = 0.825), with a minimum of 0.604 and a maximum of 0.963.

### Associations between traditional measures of disease activity and ultrasound scores

In the cross-sectional analyses, we found moderate to strong correlation between ultrasound measures such as GS and PD synovitis scores, and the following variables: TJC (*r* = 0.532, *P* <0.005 and *r* = 0.580, *P* <0.001, respectively), SJC (*r* = 0.684, *P* <0.001 and *r* = 0.606, *P* <0.001, respectively), DAS28 (*r* = 0.631, *P* <0.001 and *r* = 0.707, *P* <0.001, respectively), CRP (*r* = 0.451, *P* <0.01 and *r* = 0.463, *P* <0.005, respectively) and ESR (*r* = 0.376, *P* <0.05 and *r* = 0.428, *P* <0.01, respectively). However, there was no significant association between GS or PD tenosynovitis and the above-mentioned measures of disease activity.

### Calprotectin is associated with clinical, laboratory and ultrasound disease activity

Serum calprotectin levels were significantly correlated with SJC (*r* = 0.465, *P* <0.005), DAS28 (*r* = 0.430, *P* <0.01), ESR (*r* = 0.370, *P* <0.05) and, in particular, CRP levels (*r* = 0.629, *P* <0.001). There was no association between serum calprotectin levels and TJC, neither with GS nor PD tenosynovitis scores. Calprotectin was significantly associated with GS (*r* = 0.359, *P* <0.05) and more significantly, with PD synovitis score (*r* = 0.497, *P* <0.005) (Table 2). Calprotectin serum levels were not associated with IgM-RF or anti-CCP levels.

**Table 2:** Spearman’s rank correlation coefficients between serum calprotectin and clinical, laboratory and ultrasound parameters

Parameter	CRP	ESR	DAS28	GS syn	PD syn	GS ten	PD ten
Calprotectin	0.629**	0.370*	0.430**	0.359*	0.497**	0.192	0.210
CRP		0.636**	0.650**	0.451**	0.463**	0.314	0.314
ESR			0.659**	0.376*	0.428**	0.300	0.300
DAS28				0.631**	0.707**	0.226	0.269
GS syn					0.700**	0.252	0.267
PD syn						0.226	0.303

**Correlation significant at the 0.01 level; *correlation significant at the 0.05 level. *CRP* C - reactive protein, *ESR* erythrocyte sedimentation rate, *DAS28* 28 joint disease activity score with ESR, *GS syn* Grey scale synovitis score, *PD syn* Power Doppler synovitis score, *GS ten* Grey scale tenosynovitis score, *PD ten* Power Doppler tenosynovitis score

### The predictive value of calprotectin for ultrasound synovitis

Hierarchical multiple regression analysis was used to predict PD synovitis score by calprotectin and CRP in the first step; the effect of sex and age was controlled by adding these variables in the second step. In the univariate analyses, calprotectin was shown to be better (*β* = 0.001, *P* <0.001, *R*^2^ = 0.757) than CRP (*β* = 0.215, *P* <0.001, *R*^2^ = 0.495) at predicting PD synovitis score. For calprotectin and CRP, the increase of *R*^2^ in predicting PD synovitis score was not significantly affected by adding sex and age into the regression model (Table 3).

**Table 3:** Hierarchical multiple regression analyses predicting Power Doppler determined (PD) synovitis score by calprotectin and C-reactive protein (CRP)

	Δ *R* ^2^	*b* (Standard error)	Std. *b*	*P*
Step 1	0.757			0.000
Calprotectin		0.001 (0.00)	0.870	0.000
Step 2	0.765			0.576
Calprotectin		0.001 (0.00)	0.870	0.000
Sex		0.491 (0.67)	0.068	0.471
Age		0.019 (0.02)	0.091	0.331
Step 1	0.495			0.000
CRP		0.215 (0.04)	0.704	0.000
Step 2	0.496			0.989
CRP		0.215 (0.04)	0.701	0.000
Sex		0.022 (1.07)	0.003	0.984
Age		−0.004 (0.03)	−0.018	0.898

Note: sex was coded as 0 = males and 1 = females. *Std*. standardised

## Discussion

In this study, we demonstrated a significant association between serum calprotectin level, clinical and laboratory markers of disease activity, and ultrasound PD synovitis score in RA patients. We found that calprotectin might be a better predictor of ultrasound-determined synovial inflammation than CRP.

Calprotectin is predominantly produced at synovial inflammation sites and reflects the amount of activated synovial macrophages [13]. As a small molecule, calprotectin easily diffuses from synovial fluid and inflamed synovium into the bloodstream [11]. Correspondingly, serum calprotectin strongly correlates with that in synovial fluid and is significantly associated with acute-phase reactants and clinical disease activity [16, 40, 41], which was observed in our study. In clinical practice, however, some patients do not develop high CRP levels in spite of active disease, and there is evidence that calprotectin could be elevated even when the acute-phase reactants are normal [42, 43]. Therefore, calprotectin could provide more relevant information on disease activity than does CRP and might be an important biomarker of disease activity in RA [44].

Recently, we found that a decrease in the serum levels of calprotectin, but not CRP, was a significant predictor for improvement in the total number of swollen joints in RA [21]. To further ensure an accurate assessment of joint inflammation, we provided ultrasound measurements of disease activity in RA, which could be a more sensitive tool than a clinical examination for determining inflamed joints [45]. Several ultrasound scoring indices evaluating different joints and combinations have been developed showing good correlation with disease activity [38, 46, 47]. In this context, the German US7 score developed by Backhaus et al. includes an examination of the most frequently affected joints in RA and has been shown to be a valuable and reliable tool for the ultrasound examination of inflamed joints, which is suitable for monitoring disease activity [36]. This reduced 7-joint ultrasound score has been shown to be strongly correlated with the 78-joint score, indicating that an approach focusing on a few joints provides information on inflammatory activity in RA patients that is equivalent to that of a comprehensive ultrasound examination [48]. In agreement with several reports [25, 49], we found significant correlation between ultrasound measures, and clinical and conventional laboratory markers of disease activity. Furthermore, we demonstrated a moderate association between the levels of serum calprotectin and ultrasonography-determined synovitis in GS and, more importantly, with PD synovitis, reflecting actively inflamed joints. Our results thus support recent findings by Hammer et al., who found significant correlation between circulating calprotectin and comprehensive ultrasound assessment in 20 patients with established RA [33]. More importantly, we found that serum calprotectin might be superior to CRP in predicting ultrasound-determined synovial inflammation.

Our study has some limitations. First, the design was cross-sectional. Second, disease duration was short, and RA disease activity was relatively low, primarily because the majority of the patients were recruited from a cohort with early RA [50]. These patients initiated treatment within the first months of symptom duration, and the majority reached remission or low disease activity. Third, a relatively small number of patients were included in this study. A longitudinal study evaluating the association between calprotectin and ultrasound synovitis in patients with long-duration and active RA has already been performed [33], although the number of patients was smaller than in our study. A further weakness of our study was the participation of more ultrasonography investigators, which, however, could be considered a strong point because having additional investigators reflects routine clinical practice more accurately. Further studies on larger cohorts are needed to confirm these data.

## Conclusions

This study shows significant association between serum levels of calprotectin and clinical, laboratory, and ultrasound assessments of joint inflammation in RA patients. We suggest that calprotectin might be a valuable serological marker of RA inflammatory disease activity.

## Abbreviations

Anti-CCP: anticyclic citrullinated peptide antibody
CRP: C-reactive protein
DAS28: Disease activity score for 28 joints
ESR: erythrocyte sedimentation rate
F: female
GS syn: Grey scale synovitis
GS ten: Grey scale tenosynovitis
M: male
PD syn: Power Doppler synovitis
PD ten: Power Doppler tenosynovitis
RA: rheumatoid arthritis
RF: rheumatoid factor
SJC: swollen joint count
TJC: tender joint count

## References

[1] Kitas GD, Gabriel SE (2011). Cardiovascular disease in rheumatoid arthritis: state of the art and future perspectives. Ann Rheum Dis..

[2] Smolen JS, Aletaha D, Bijlsma JW, Breedveld FC, Boumpas D, Burmester G (2010). Treating rheumatoid arthritis to target: recommendations of an international task force. Ann Rheum Dis..

[3] Pincus T, Castrejon I, Bergman MJ, Yazici Y (2012). Treat-to-target: not as simple as it appears. Clin Exp Rheumatol..

[4] van der Heijde DM, Hof MA V ’t, van Riel PL, Theunisse LA, Lubberts EW, van Leeuwen MA (1990). Judging disease activity in clinical practice in rheumatoid arthritis: first step in the development of a disease activity score. Ann Rheum Dis.

[5] Prevoo ML, Hof MA V 't, Kuper HH, van Leeuwen MA, van de Putte LB, van Riel PL (1995). Modified disease activity scores that include twenty-eight-joint counts. Development and validation in a prospective longitudinal study of patients with rheumatoid arthritis. Arthritis Rheum.

[6] Smolen JS, Breedveld FC, Schiff MH, Kalden JR, Emery P, Eberl G (2003). A simplified disease activity index for rheumatoid arthritis for use in clinical practice. Rheumatology (Oxford).

[7] Aletaha D, Nell VP, Stamm T, Uffmann M, Pflugbeil S, Machold K (2005). Acute phase reactants add little to composite disease activity indices for rheumatoid arthritis: validation of a clinical activity score. Arthritis Res Ther..

[8] Foell D, Roth J (2004). Proinflammatory S100 proteins in arthritis and autoimmune disease. Arthritis Rheum..

[9] Odink K, Cerletti N, Bruggen J, Clerc RG, Tarcsay L, Zwadlo G (1987). Two calcium-binding proteins in infiltrate macrophages of rheumatoid arthritis. Nature..

[10] Wilkinson MM, Busuttil A, Hayward C, Brock DJ, Dorin JR, Van Heyningen V (1988). Expression pattern of two related cystic fibrosis-associated calcium-binding proteins in normal and abnormal tissues. J Cell Sci..

[11] Dale I, Fagerhol MK, Naesgaard I (1983). Purification and partial characterization of a highly immunogenic human leukocyte protein, the L1 antigen. Eur J Biochem..

[12] Hetey L, Doring E, Penke B (1987). Influence of cholecystokinin on the synaptosomal dopamine uptake in the nucleus accumbens of rats. Neurochem Int..

[13] Johne B, Fagerhol MK, Lyberg T, Prydz H, Brandtzaeg P, Naess-Andresen CF (1997). Functional and clinical aspects of the myelomonocyte protein calprotectin. Mol Pathol..

[14] Berntzen HB, Fagerhol MK, Ostensen M, Mowinckel P, Hoyeraal HM (1991). The L1 protein as a new indicator of inflammatory activity in patients with juvenile rheumatoid arthritis. J Rheumatol..

[15] Berntzen HB, Munthe E, Fagerhol MK (1989). A longitudinal study of the leukocyte protein L1 as an indicator of disease activity in patients with rheumatoid arthritis. J Rheumatol..

[16] Brun JG, Haga HJ, Boe E, Kallay I, Lekven C, Berntzen HB (1992). Calprotectin in patients with rheumatoid arthritis: relation to clinical and laboratory variables of disease activity. J Rheumatol..

[17] Hammer HB, Kvien TK, Glennas A, Melby K (1995). A longitudinal study of calprotectin as an inflammatory marker in patients with reactive arthritis. Clin Exp Rheumatol..

[18] Frosch M, Vogl T, Seeliger S, Wulffraat N, Kuis W, Viemann D (2003). Expression of myeloid-related proteins 8 and 14 in systemic-onset juvenile rheumatoid arthritis. Arthritis Rheum..

[19] Haga HJ, Brun JG, Berntzen HB, Cervera R, Khamashta M, Hughes GR (1993). Calprotectin in patients with systemic lupus erythematosus: relation to clinical and laboratory parameters of disease activity. Lupus..

[20] Youssef P, Roth J, Frosch M, Costello P, Fitzgerald O, Sorg C (1999). Expression of myeloid related proteins (MRP) 8 and 14 and the MRP8/14 heterodimer in rheumatoid arthritis synovial membrane. J Rheumatol..

[21] Andres Cerezo L, Mann H, Pecha O, Plestilova L, Pavelka K, Vencovsky J (2011). Decreases in serum levels of S100A8/9 (calprotectin) correlate with improvements in total swollen joint count in patients with recent-onset rheumatoid arthritis. Arthritis Res Ther..

[22] Hammer HB, Odegard S, Syversen SW, Landewe R, van der Heijde D, Uhlig T (2010). Calprotectin (a major S100 leucocyte protein) predicts 10-year radiographic progression in patients with rheumatoid arthritis. Ann Rheum Dis..

[23] Wakefield RJ, Goh E, Conaghan PG, Karim Z, Emery P (2003). Musculoskeletal ultrasonography in Europe: results of a rheumatologist-based survey at a EULAR meeting. Rheumatology (Oxford).

[24] Backhaus M, Kamradt T, Sandrock D, Loreck D, Fritz J, Wolf KJ (1999). Arthritis of the finger joints: a comprehensive approach comparing conventional radiography, scintigraphy, ultrasound, and contrast-enhanced magnetic resonance imaging. Arthritis Rheum..

[25] Naredo E, Bonilla G, Gamero F, Uson J, Carmona L, Laffon A (2005). Assessment of inflammatory activity in rheumatoid arthritis: a comparative study of clinical evaluation with grey scale and power Doppler ultrasonography. Ann Rheum Dis..

[26] Szkudlarek M, Court-Payen M, Jacobsen S, Klarlund M, Thomsen HS, Ostergaard M (2003). Interobserver agreement in ultrasonography of the finger and toe joints in rheumatoid arthritis. Arthritis Rheum..

[27] Scire CA, Montecucco C, Codullo V, Epis O, Todoerti M, Caporali R (2009). Ultrasonographic evaluation of joint involvement in early rheumatoid arthritis in clinical remission: power Doppler signal predicts short-term relapse. Rheumatology (Oxford).

[28] Brown AK, Quinn MA, Karim Z, Conaghan PG, Peterfy CG, Hensor E (2006). Presence of significant synovitis in rheumatoid arthritis patients with disease-modifying antirheumatic drug-induced clinical remission: evidence from an imaging study may explain structural progression. Arthritis Rheum..

[29] Saleem B, Brown AK, Keen H, Nizam S, Freeston J, Wakefield R (2011). Should imaging be a component of rheumatoid arthritis remission criteria? A comparison between traditional and modified composite remission scores and imaging assessments. Ann Rheum Dis..

[30] Wakefield RJ, Freeston JE, Hensor EM, Bryer D, Quinn MA, Emery P (2007). Delay in imaging versus clinical response: a rationale for prolonged treatment with anti-tumor necrosis factor medication in early rheumatoid arthritis. Arthritis Rheum..

[31] Ozgocmen S, Ozdemir H, Kiris A, Bozgeyik Z, Ardicoglu O (2008). Clinical evaluation and power Doppler sonography in rheumatoid arthritis: evidence for ongoing synovial inflammation in clinical remission. South Med J..

[32] Saleem B, Brown AK, Quinn M, Karim Z, Hensor EM, Conaghan P (2012). Can flare be predicted in DMARD treated RA patients in remission, and is it important? A cohort study. Ann Rheum Dis..

[33] Hammer HB, Fagerhol MK, Wien TN, Kvien TK (2011). The soluble biomarker calprotectin (an S100 protein) is associated to ultrasonographic synovitis scores and is sensitive to change in patients with rheumatoid arthritis treated with adalimumab. Arthritis Res Ther..

[34] Arnett FC, Edworthy SM, Bloch DA, McShane DJ, Fries JF, Cooper NS (1988). The American Rheumatism Association 1987 revised criteria for the classification of rheumatoid arthritis. Arthritis Rheum..

[35] Aletaha D, Neogi T, Silman AJ, Funovits J, Felson DT, Bingham CO (2010). Rheumatoid arthritis classification criteria: an American College of Rheumatology/European League Against Rheumatism collaborative initiative. Arthritis Rheum.

[36] Backhaus M, Ohrndorf S, Kellner H, Strunk J, Backhaus TM, Hartung W (2009). Evaluation of a novel 7-joint ultrasound score in daily rheumatologic practice: a pilot project. Arthritis Rheum..

[37] Backhaus MWS, Ohrndorf S, Alraqi S, Crowther S, Dhillon S, Dhindsa N (2013). Can satisfactory reliability of the 7-joint ultrasound score be attained by inexperienced Readers In a single calibration exercise? results from the biodam program. [abstract]. Arthritis Rheum..

[38] Scheel AK, Hermann KG, Kahler E, Pasewaldt D, Fritz J, Hamm B (2005). A novel ultrasonographic synovitis scoring system suitable for analyzing finger joint inflammation in rheumatoid arthritis. Arthritis Rheum..

[39] Landis JR, Koch GG (1977). The measurement of observer agreement for categorical data. Biometrics..

[40] Garcia-Arias M, Pascual-Salcedo D, Ramiro S, Ueberschlag ME, Jermann TM, Cara C (2013). Calprotectin in rheumatoid arthritis: association with disease activity in a cross-sectional and a longitudinal cohort. Mol Diagn Ther..

[41] Hammer HB, Odegard S, Fagerhol MK, Landewe R, van der Heijde D, Uhlig T (2007). Calprotectin (a major leucocyte protein) is strongly and independently correlated with joint inflammation and damage in rheumatoid arthritis. Ann Rheum Dis..

[42] Foell D, Frosch M, Schulze zur Wiesch A, Vogl T, Sorg C, Roth J (2004). Methotrexate treatment in juvenile idiopathic arthritis: when is the right time to stop?. Ann Rheum Dis.

[43] Foell D, Wulffraat N, Wedderburn LR, Wittkowski H, Frosch M, Gerss J (2010). Methotrexate withdrawal at 6 vs 12 months in juvenile idiopathic arthritis in remission: a randomized clinical trial. JAMA..

[44] Senolt L (2012). Calprotectin (a S100 protein) as a sensitive biomarker for rheumatoid arthritis: new perspectives for an old finding. Int J Clin Rheumatol..

[45] Colebatch AN, Edwards CJ, Ostergaard M, van der Heijde D, Balint PV, D’Agostino MA (2013). EULAR recommendations for the use of imaging of the joints in the clinical management of rheumatoid arthritis. Ann Rheum Dis..

[46] Naredo E, Rodriguez M, Campos C, Rodriguez-Heredia JM, Medina JA, Giner E (2008). Validity, reproducibility, and responsiveness of a twelve-joint simplified power doppler ultrasonographic assessment of joint inflammation in rheumatoid arthritis. Arthritis Rheum..

[47] Perricone C, Ceccarelli F, Modesti M, Vavala C, Di Franco M, Valesini G (2012). The 6-joint ultrasonographic assessment: a valid, sensitive-to-change and feasible method for evaluating joint inflammation in RA. Rheumatology (Oxford).

[48] Hammer HB, Kvien TK (2011). Comparisons of 7- to 78-joint ultrasonography scores: all different joint combinations show equal response to adalimumab treatment in patients with rheumatoid arthritis. Arthritis Res Ther..

[49] Hameed B, Pilcher J, Heron C, Kiely PD (2008). The relation between composite ultrasound measures and the DAS28 score, its components and acute phase markers in adult RA. Rheumatology (Oxford).

[50] Senolt L, Cerezo LA, Sumova B, Pecha O, Plestilova L, Forejtova S (2015). High levels of metastasis-inducing S100A4 protein and treatment outcome in early rheumatoid arthritis: data from the PERAC cohort. Biomarkers..

